# New forms of internationalisation? The impact of Netflix in Australia

**DOI:** 10.1177/1329878X20941173

**Published:** 2020-11

**Authors:** Stuart Cunningham, Alexa Scarlata

**Affiliations:** Queensland University of Technology, Australia; RMIT University, Australia; University of Melbourne, Australia

**Keywords:** Australian screen industry, COVID-19, internationalisation in screen industries, multinational SVOD services, Netflix

## Abstract

This article examines the impact of multinational subscription video-on-demand (SVOD) services in Australia, noting the degree to which a stalled policy response to the challenge of unregulated SVOD services has been shaken up by coronavirus disease 2019 (COVID-19). We look at the phenomenon from a screen-ecological perspective – where dynamics of consumption, reviewing, production and regulation are interdependently and often contradictorily in play. We examine how these diverse, sometimes conflicted, perspectives can be approached as responding to new forms of internationalisation presented principally by the operations of Netflix in Australia (Amazon Prime Video, Disney+ and Apple TV+ are also mentioned when relevant). This article is part of a larger project (ARC Discovery DP190100978 Internet-Distributed Television: Cultural, Industrial and Policy Dynamics, chief investigators Ramon Lobato, Amanda Lotz, Stuart Cunningham) studying the cultural, industrial and policy dynamics of multinational SVOD globally and in situ locally.

## A new form of internationalisation?

Netflix Chief Content Officer, Ted Sarandos, has said that the company is ‘not trying to make more Hollywood content for the world. Rather, a big part of the strategy is to bring content from different countries to a worldwide audience’ (as cited by Spangler, 2018). When asked about Netflix’s role in exporting American values, CEO Reed Hastings (2019) preferred to describe what they do as ‘mutual sharing. We serve 190 counties’, he said, ‘and we try to think of them all on the same footing’. These carefully crafted statements from the US tech titan are worth contemplating. Netflix’s two top executives position the company as a kind of screen-cultural traffic cop, intermediating a new multilateralism and pluriform interchange of much more heterogeneous content than any previous account of media imperialism, cultural imperialism, globalisation or glocalisation has offered.

We agree that Sarandos’ articulation of Netflix’s status as a place for the world’s content is being borne out in its rapidly evolving Originals commissioning strategy. With more than 60% of its subscribers outside the United States (Netflix Investors, 2019), it is clear that ‘international is where the big opportunities for growth lie’ (Clarke, 2019a). To support this, Netflix has been setting up regional headquarters, production facilities, outposts and policy teams (including in Australia) and scaling their investments to match their growth (Luegenbiehl, as cited by Clarke, 2019b). Without a doubt, Netflix is now the largest multilingual, multinational commissioner of original scripted screen content the world has ever seen.

However, while this suggests a fundamentally post-cultural hegemonic strategy, Netflix catalogues of licenced content are a different matter. The European Audiovisual Observatory has suggested that the average Netflix catalogue in European countries contains content from more than 50 different countries, but around half the titles are from the United States (Grece, 2018). The European Union’s (EU) member countries have until late 2020 to incorporate the 30% content quota obligations approved by the European Parliament in 2018, but the details of whether that percentage will refer to hours of content, the number of titles, or some combination of these factors remains unclear (Vourlias, 2020). Annual research into Australian content in the local Netflix offer has found that since 2017 local content has constituted at most 2.5% of the catalogue – and, more recently, only 1.7% (Lobato and Scarlata, 2019). There is more Australian content in the US catalogue than the Australian catalogue! Of course, actual viewing is of ultimate importance, but catalogue data matters as they stand in for largely unknown viewing data from SVODs, and it is the menu from which viewing choices are perforce made.

## Brand power and the politics of consumption

SVOD has had a remarkably rapid take-up in Australia, overtaking the monopoly pay-TV provider Foxtel within 18 months of launch (Roy Morgan, 2016) to reach more than half the population in 5 years (ACMA, 2020; Roy Morgan, 2019). Netflix has rushed to market leader in non-linear TV, with an estimated 12.2 million Australians now having access to a Netflix subscription in their household (Roy Morgan, 2020). Australia’s embrace of Netflix is demonstrated by its ranking as the country with the second highest penetration rate (i.e. the percentage of the population who subscribes to the service; Ampere Analysis, 2019). The highest on this measure is Canada, where Netflix has been available for twice as long as Australia. With Amazon Prime Video already firmly ensconced, Apple’s popularity in the Australian smartphone market (Kidman, 2020) and offer of a year of free Apple TV+ with the purchase of a new device, and Disney’s selection of Australia as one of their first four international markets for their direct-to-consumer streaming launches in November 2019 (Ampere Analysis, 2019) presage continuing rapid foreign transformational impact on the local television space.

Netflix’s disruption of the subscription model in Australian TV has seen the pay-TV sector sufficiently challenged for the monopoly service, Foxtel, to be forced to unbundle and rebrand as a multiplatform service (McDonnell, 2019). Constantly pivoting to stay viable despite persistently static reach figures, it has integrated Netflix into an ‘all in one place’ offer, ‘The New Foxtel Experience’.

Netflix’s success is a testament to the power of the company’s brand – the fastest-growing US brand in 2019 (Spangler, 2019a) – and to a range of consumer or demand-side factors, including a high-quality user experience (Spangler, 2019b), a relatively consistent price point that starts competitively low (Masige, 2019), the entrenchment of the service into our lexicon (Rickett, 2015) and popular conceptions of viewing (‘Netflix and chill’), and an overarching emphasis on exclusive original programming and regular content acquisitions that are often touted as innovating in screen content (e.g. *Black Mirror: Bandersnatch, The Irishman*), created in part for a ‘lost generation’ of Millennial and younger viewers (Brownlow as cited by Cunningham, 2018/19).

Critical media scholar Graeme Turner (2018) has written strongly about the ‘game changer’ that is Netflix’s reconfiguration of the Australian television market. To be sure, the markets into which Netflix has penetrated deepest are mostly the Anglosphere, where the acceptance of what remains still a largely English-language, US-dominated offer, is highest (Ampere Analysis, 2019). But it also points to fundamental weaknesses in the Australian television system and ‘pent up’ demand (about a quarter of a million VPN-based Netflix users *before* launch in 2015 (Lobato and Ewing, 2014) in the context of the classic Silicon Valley disruptive play: enter a highly regulated market with a low-priced alternative (in this case, to pay-TV provider Foxtel) based on technological solutions of genuine benefit to consumers (providing a high-quality user experience) that can be scaled rapidly with a minimum commitment to the sunk costs of hard infrastructure assets.

Turner (2019a, 2019b) is also right to point to these remarkable consumption phenomena throwing down the challenge to scholarship to reengage with audience studies and ‘cultures of use’, thus casting some light, beyond clumsy clichés like binge viewing, on what is driving such change at the household level. The ‘Netflix effect’, Turner (2019a: 223) argues, requires nothing less than the ‘need to reconsider the adequacy of the currently preferred methodologies for analysing media consumption’. The call for this kind of research is especially pertinent as another key page in the Silicon Valley playbook is to establish unacceptably high levels of information asymmetry, releasing very little consumption data, even to their partner producers.

One thing such change may be seen to have challenged – but we would argue has not undermined – is the propensity Australian audiences have to consume Australian content. The ‘Hollywood + Australian content’ strategy of the Australian-based and Australian-owned streamer Stan, as described by CEO Mike Sneesby (2019), has seen them establish a significant foothold in the local market, reaching an estimated 3.7 million Australians (Roy Morgan, 2020) from a base of 1.8 million subscriptions (Meade, 2020). While Stan will never compete with the brand power of Netflix, its consistent growth, partnerships with traditional US distributors, and an emphasis on local production have defied initial expectations of the local broadcaster-owned SVOD, upon which Nine is increasingly relying to ride out this period of radical market uncertainty (Mediaweek, 2020). It appears that Stan’s latest strategy to respond to the entry of Apple TV+ and Disney+ (with the consequent loss of significant content packages) has been to lean into ‘homegrown’ branding even further. Recent advertising has Stan describing itself as ‘Australia’s unrivalled home of original productions’ (Stan Australia, 2019), a title confirmed by the fact that while Australian content in the Netflix AU catalogue has remained at or below 2% since its launch, local titles have made up 9%–11% of Stan’s library since 2017 (Lobato and Scarlata, 2019).

One potentially useful response to Turner’s call for a ‘cultures of use’ approach, in the absence of at least a beginning point with audience data and, as Turner notes, no extant Australian qualitative academic studies, is to look at how professional reviewers are shaping Australian consumption of SVOD content.

## Cultural blind spots: Australian originals on Netflix and Stan

Perhaps the most culturally intriguing of all the impacts of multinational streaming services on the Australian screen ecology is the local struggle to understand, not to say appreciate, the new dynamics of internationalisation. Netflix’s *cultural* profile as a foreign, specifically American, entity is starkly clear in the analytical frames being used by professional reviewers looking to make sense of the corpuses of Australian originals being produced by Netflix and its main local competitor, Stan. Cultural criticism and reviewing of these corpuses display a resumption of the old opposition between Dermody and Jacka’s (1987) ‘discourses of nationalism’ and ‘discourses of internationalism’. Whereas Stan’s Australian originals are reviewed as familiar extensions of well-understood local genres and routines of representation, Netflix’s major commissions so far, *Pine Gap, Tidelands* and *Lunatics*, have been met with bemusement, befuddlement and excoriation.

The critical response to Stan’s originals has been almost uniformly warm and supportive, even gushing. Their comedy series – *No Activity* and *The Other Guy* – were overwhelmingly described as familiar, but fresh. Largely improvised *No Activity* was a ‘sly delight’ that delivered ‘Australian farce at its finest’ (Bastow, 2016). *The Other Guy*, a semi-autobiographical comedy-drama starring comedian Matt Okine and ‘distinctly set in Sydney’s inner west’ (Groves, 2019) was a ‘refreshingly casual’ but ‘ambitious’ look at ‘who we are’ (Moran, 2017). Shows like these should, it was put forth, ‘put paid to the idea that online streaming is only a graveyard for the sitcoms of yesteryear’ (Burke, 2015). Stan’s first forays into original television drama benefitted significantly from local familiarity with their well-established and popular film source material. The series adaptation of *Wolf Creek* was considered a ‘very Australian example of the horror genre’ (Burke, 2017), that looked ‘like a million blood-stained bucks’ and boasted ‘considerable gnarly thrills’ (Buckmaster, 2016). The remake of the 1990s classic *Romper Stomper* premiered on New Year’s Day 2018 and was deemed a timely and accurate representation of the times; a series that ‘feels achingly real and relevant’ (Buckmaster, 2017); ‘it’s risky stuff, and for the most part it works’ (Quinn, 2017). The SVOD’s first original film, *The Second*, summoned in Buckmaster (2018b) ‘equal feelings of awe and incredulity’. Stan’s original concept, *Bloom*, released in January 2019, was a ‘brilliant and tragic’ ‘gothic fairy-tale’ anchored on a fantastical premise that was, again, ‘ambitious’ (Quinn, 2018).

Stan publicly welcomed the launch of Apple TV+ and Disney+ at the end of 2019 (Blackiston, 2019b) with a big summer offer of four Australian originals. Pedestrian described the second season of *The Other Guy* as the ‘most legit TV show about Aussie millennials’ (Galea, 2019); for *The Guardian*, the ‘sophisticated comedic work’ raised some ‘uncomfortable – and dizzying questions’ (Valentish, 2020). Science-fiction thriller *The Commons*, Stan’s most expensive show to date at approximately AU$25 million (Ma, 2019a), envisioned a climate crisis-devastated Australia. Released on Christmas Day to a drought, dust storm and fire-ravaged nation, it was described as ‘unnervingly prescient’ (Ma, 2019a) and ‘grounded and gritty’ (Morris, 2019). Buckmaster (2019b) deemed it ‘eerily plausible and uncomfortably timely’: ‘By contemplating big, meaty subjects … within a distinctively Australian context, with plenty of recognisable (CGI-enhanced shots) of Sydney, we are forced to contemplate how our own communities and cities might look going forward’.

The ‘character-driven and moody Tasmanian noir’ *The Gloaming* apparently offered ‘an aesthetic so eye-watering it makes plaudits such as “evocative” or “painterly” seem manifestly inadequate’ (Buckmaster, 2019c). The show was lauded as the kind of ‘ambitious’ and ‘gutsy’ Australian series being largely abandoned by commercial free-to-air networks (Ma, 2020). Stan also made a ‘hefty investment’ (Groves, 2020) in Justin Kurzel’s *True History of the Kelly Gang* to secure the feature for release on the streaming service on Australia Day 2020, after a limited cinema window. While the accuracy of the film’s portrayal of the legendary Australian bushranger was called into question (Gaunson, 2020), Stan benefitted from the publicity generated by its international cast, Toronto Film Festival premiere and the largely positive critical reception, both locally (Boyd, 2020; Bradshaw, 2019; Galea, 2020) and internationally (Lodge, 2019; Rooney, 2019).

In stark contrast, reviewers have struggled to productively come to grips with Netflix’s Australian originals. The commissioning of the ABC/Netflix co-production *Pine Gap* saw high hopes that this ‘timely’ thriller (Idato, 2017) based on a concept ‘shrouded in intrigue for many Australians’ would be ‘just the beginning’ of a slate of new content production (Slessor and Bogle, 2018). However, upon release, it was thoroughly panned. Buckmaster (2018a) described it as ‘less a spy drama than an attempt to cure insomnia’ and awarded it a solitary star. LaMarco (2018) lamented that while the setting is a ‘unique one and could be interesting to American audiences not familiar with the territory’, that is where the reasons to watch the ‘dull and sluggish attempt at a thriller’ end. Razer (2018) wrote that *Pine Gap* was flagrant propaganda of the ‘state-approved sentiment we see on ABC TV news constantly: China is “inscrutable”; the US is imperfect but basically decent; Muslim terrorists are the very worst kind of terrorist. Oh, and this old favourite: Aboriginal people are deeply spiritual’. With its ‘laborious exposition’ and lazy dialogue, it insulted in its representations (Razer, 2018).

The response to Netflix’s first fully commissioned Australian original, *Tidelands*, was largely befuddlement, and witnessed the recrudescence of the cultural cringe. ‘*Tidelands* is an embarrassing mess’ and ‘not the show Australia deserves to represent us on the US streaming service’, wrote (Ma, 2018). While the supernatural crime thriller was undeniably pretty – ‘all sun-kissed glistening bodies and perfect beach hair’ – Ma deemed the plot confusing, the writing weak, the performances lightweight, and the show ‘strangely boring’ overall (2018). *The Conversation* found the series ‘contains many narratives’ and as a result suffers from ‘something of an identity crisis … The result is a tangled confusion of storylines … [where] nudity and sex are used as temporary resolutions to sub-plots, distracting from more major plotlines’ (Turner, 2018a). Fairfax reviewer Houston (2018), in classical cringe mode, began her review with ‘What will the international audience for whom it’s clearly intended make of Tidelands?’.

However, this response was measured when compared with the critical vitriol Chris Lilley’s Netflix original, *Lunatics*, attracted. Lilley’s satire is perennially controversial and attracts inevitable backlash. *Lunatics’* use of blackface saw some fans cancel their subscriptions (Nsenduluka, 2019) and take to Twitter to boycott the streaming platform (Powell, 2019) and ultimately, in the light of 2020’s huge upswell of Black Lives Matter protests, saw Netflix withdraw all four of Lilley’s shows from its Australian catalogue (Boseley, 2020). But it should also be noted that the series rated highly with audiences in Australia according to Netflix’s end of year most popular lists. Locally, it ranked fifth in the top 10 most popular releases of 2019 and second in the list of most popular series (White and Kanter, 2019).

Lilley’s first series in almost 5 years was met with critical indifference – ‘the worst reaction you could elicit in comedy’ (Ma, 2019b) – and excoriation. For Ma, it wasn’t just that he hadn’t moved on from the ‘same schtick’ that brought him early acclaim. Instead, with *Lunatics* he seemed to have ‘less insight and discipline’, the freedom of Netflix’s episode lengths facilitating ‘fundamental structural issues’, with ‘sprawling’ sketches that ‘overstayed their welcome’ (Ma, 2019b). Heritage (2019) concluded that ‘the problem isn’t that the series is cruel … It’s that it is boring. The trick has stopped working. The whole thing just feels rote’. Buckmaster doubled down: he considered Lilley and his humour ‘a relic from an older, more primitive time’, and questioned the SVOD’s decision to commission the series: ‘Just when it appeared no TV network in Australia would work with him again, along came Netflix (talk about not reading the room) … *Lunatics* is a disaster: not just unfunny and problematic, but boring and utterly unrelenting’ (2019a).

Of course, *de gustibus non est disputandum* – you can’t argue with taste. Maybe it just so happens that all these Netflix originals are just awful and all of Stan’s work is simply outstanding. And tomorrow it will all turn around because new work will change the evidence base. But one fundamental thing cultural studies has taught us is that taste is socially and culturally constructed and reproduced, so we will proceed to interpret this evidence base as a social and cultural construct that goes to the heart of how Australian industry, culture, consumers and policy respond to new forms of internationalisation presented by the operations of Netflix in Australia.

The remarkably different cultural assumptions in the reviewing of these two bodies of work reflect old, powerful discourses of ‘good’ nationalism versus ‘bad’ internationalism that date from the 1970s. Susan Dermody and Elizabeth Jacka (1987), seeking to account for the dynamic conflictual structure of the renascent Australian film industry in the 1970s and 1980s, contrasted Industries 1 (culturally specific) and 2 (blandly international). This foundational analysis has been partially contemporised by Deb Verhoeven (2014). She argued that, since the 1980s, ‘Industry 3’ (filmmaking that is both national and international) and ‘Industry 4’ have emerged. Industry 4 is ‘characterised by the adoption of new methodologies for producing and distributing content afforded by the digitisation of the screen industries’ (p. 163). Some of the characteristics of Verhoeven’s Industry 4 presage the impact of SVODs, especially her delineation of ‘global niche audiences’ ‘defined by interest rather than geo-political allegiance’. Both her Industry 3 and 4 declare a strong realignment of the national–international opposition (p. 164).

The radically different critical response to Stan’s and Netflix’s originals belies the reality that Netflix’s internationalism cannot be read through the old binaries of Industries 1 and 2, while it also exemplifies a form of Industry 4. Nor can it be put down to Stan simply commissioning better quality product. Producers of Netflix’s originals (such as Screentime and Hoodlum) are experienced and indeed industry leaders. And it cannot only be attributed to Netflix’s global, and Stan’s local, audience either – Stan’s originals have been aggressively and quite successfully sold into multiple international markets on the traditional model of first release locally, then sell internationally.

Whether Netflix fully funds or co-funds its ‘Originals’ (Ball, 2018), its investment decisions are always based on a multiple-territory strategy. It invests in content produced to occupy verticals, or genres, such as millennial supernaturalism (*Tidelands*) – which Netflix may not have invented but has done much to popularise globally – or very edgy or controversial comedy (*Lunatics*). There is a thriving genre of Netflix originals that trade on international political and conspiracy dramas, into which *Pine Gap* fits neatly. These genre categories appeal to transnational audience cohorts that could never be restricted to a single market for them to be a viable commission.

Netflix’s internationalism in these instances is not about so-called ‘runaway’ production – pretending that the locations are somewhere else. Nor does it look like international co-production, where typically the storylines are designed to take characters from country to country to appeal to audiences in specific locales and satisfy multiple national funding agencies.

Netflix’s internationalism is not concerned with downplaying Australianness; indeed, it can double down on it when it suits the marketability of the content. Some of *Tidelands’* marketing showcased Queensland in ‘all its striking coastal and inland glory’ (Hoodlum principal Nathan Mayfield, as cited in Enker, 2018). While strong tourism outcomes were central to the financial support of Screen Queensland (2017), the ‘spectacle’ of the Moreton Bay region was enthusiastically embraced by Netflix as well. (Its featurette, ‘Welcome to Orphelin Bay’ – released as part of the show’s advertising campaign – appears, for the most part, like a tourism ad.) Netflix also offers significant international exposure opportunities for content and performers whose material would be unlikely to appear on broadcast television (the best Australian example is Hannah Gadsby’s *Nanette* and *Douglas*). Indeed, Anna Potter (2018) even argues that Netflix’s kids’ product is sometimes more Australian than local broadcaster-commissioned product.

## Production opportunity and threat

Given the warm embrace extended by Australian consumers to multinational SVODs, stark in comparison with the cold shoulder shown to Netflix by professional reviewers, it is important to stress the cultural innovation and industry stimulus that disruptive forces such as Netflix have visited on production environments, including Australia’s. In general, international SVODs have provided another distribution and funding source; picked up and revived series dumped by broadcasters (Blackiston, 2019a); strengthened or reinvented genres (e.g. anime, horror, sci-fi, genres often addressed to teenage viewers); addressed underserved demographics, particularly Millennials; contributed to more cosmopolitan popular culture consumption; and provided a global platform for niche interests which would rarely get through broadcaster gatekeepers. As Lotz (2020) has shown, Netflix’s fundamental strategy is to transact with multinational subscribers to pursue distinctive content strategies, in particular, programming for taste clusters too small to effectively form a viable market for services limited by national reach or nation-based audience evaluation.

For the production industry, SVOD services have provided new funding opportunities. The *Screen Australia Drama Report* (2019) Drama Report shows that, in 2017–2018 and 2018–2019, investment in drama programmes first released online equalled or surpassed those on pay-TV. It is important to contextualise spending on titles that have premiered online. This still constitutes a relatively small slice of the overall production pie, and for Screen Australia’s purposes includes content first released on all forms of online video-on-demand (Broadcast (BVOD), Advertising (AVOD) and Transactional (TVOD), as well as SVOD). This should be contrasted with the startling claim from the Motion Picture Association (MPA), which represents Disney, Netflix, Paramount, Sony, Universal and Warner Bros in the Asia-Pacific region, that SVOD commissions or co-productions in Australia ‘already exceed the investment in original drama made by commercial broadcasters without being required to do so by regulation’ (Fernandes, 2019: 3). This problematically includes any local production or co-production that has graced an SVOD in some capacity both here and abroad.

However, it is important to acknowledge that budgets for international SVOD commissions are significantly higher than what producers can expect in the massively constrained local environment threatened by broadcasters’ haemorrhaging of advertising revenue and – yes – the migration of eyeballs to SVODs. It also throws an ironic light on Minister Fletcher’s (2020) allusion that new quality benchmarks have been established by SVODs. Significantly higher budgets are very attractive to those who receive them, although that also means that cost structures across the industry can rise.

But perhaps, by volume, the most significant production stimulus that the arrival of foreign SVODs has caused has been Fairfax/Nine’s (now Nine) pre-emptive response to the imminent arrival of Netflix – the establishment of Stan. Stan was the first streaming platform to commission a locally produced television series (*No Activity*, 2015). Stan’s catalogue now contains 17 locally produced originals: two feature films, nine television series/specials and six stand-up comedy specials. Despite the fact that a large part of their annual AU$55 million originals production budget comes from international distribution (Sneesby, 2019), Stan claims that their titles are being produced with a specifically Australian audience in mind (Duke, 2018); that the business has furthered itself in Australia by filling the gaps left by multinational SVODs (Sneesby as cited in Blackiston, 2019b). Since 2018, partnerships with Victorian and Queensland state screening bodies on drama and comedy development funds have sought to unearth new local writing talent and support practitioners at all stages of production. This traditional developmental model contrasts with Netflix’s propensity to contract only with experienced producers.

Opportunity and threat come hand in hand for producers. Significantly higher budgets offered by multinational SVODs come with business practices shared with other Silicon Valley platforms: a ‘black box’ approach to deal structures and data, raising issues of transparency, accountability, fairness and terms of trade. Nondisclosure agreements, extremely selective data release and reports of a take it or leave it approach to negotiating deal structures make Netflix – despite its significant innovations and considerable commitment to quality production expenditure – a potentially bad actor in the screen production industry.

But perhaps the greatest Faustian bargain is the one that suggests that, without regulatory reform, the more producers sign with SVODs, the more they may unintentionally weaken the current settlement around Australian content regulation. The production industry depends on a stable regulatory environment focussed on the so-called ‘market failure’ genres that are most vulnerable to substitution because of their cost. It is very important to note that there is a natural fit between SVOD key genres and the core market failure genres that key commercial TV content regulation is directed at: quality scripted drama, comedy, kids and documentary. Netflix specialises in these genres (as to varying degrees do other multinational SVODs), while free to air broadcasting is increasingly specialising in light entertainment and reality, with YouTube the place for user-generated arts, performance and music (AlphaBeta, 2016). The substitution effect of the increased consumption of these genres on SVOD services means that, if there is no move to incorporate SVODs into the regulatory framework, the disparity between domestic incumbents’ regulatory burden and SVODs will grow and will strengthen the pressure brought to bear on the policy system and government by broadcasters and pay-TV for deregulation.

## To regulate or not to regulate

The regulatory pressures have now come to a head. The remarkably rapid uptake of Netflix has added to the financial and viewership woes of the commercial broadcasters, which most recently have been assailed on the advertising side by Google and Facebook, and on the attention economy side by the SVODs. Tim Worner, the then-head of leading network Seven, might have looked quixotic in 2018 in trying to tie a crisis in entertainment for the commercial broadcasters to the crisis in journalism, appealing to the competition regulator to include entertainment in its remit in the recent Australian Competition and Consumer Commission (ACCC) Digital Platforms Inquiry (Seven West Media, 2018). Now, the strategy is much clearer.

The commercial networks have been protected and assisted by government in successive waves over decades. From a consumer and political perspective, they may be too central to everyday life to fail. Government has mandated that the ACMA cannot grant more than three commercial broadcast licences. In 2017, their licence fees were abolished and replaced with reduced spectrum usage fees. Anti-syphoning rules protect their access to premium sport. They can acquit Australian content obligations using New Zealand content. Until now, though, governments have broadly held the line on Australian content regulation.

That changed, dramatically, in April 2020. COVID-19 has been the spur for the Morrison government to ‘temporarily’ suspend the market failure genre regulations (while retaining the so-called transmission quota). The move is uncomfortably close to Seven West Media’s call on the federal government to take ‘immediate action’ and abandon local content quotas. Following a AU$66 million half-year loss at the end of 2019, the broadcaster revealed their plan to breach their licencing requirements in 2021 by halting the ‘unsustainable’ production of local children’s content that is ‘further handicap[ping them] against unregulated, foreign digital platforms’ (Samios and Hunter, 2020).

SVOD success is the latest factor leeching the lifeblood out of the regulations protecting the market failure genre. Nine Entertainment announced AU$100 million in cuts to their broadcast service, intending to concentrate on the content that is proving more profitable in the digital era – namely, sport, news, current affairs and local reality (Meade, 2020).

The matter of regulating SVOD services is vexed. Such services were established in various countries, including Australia, not as television services but as telecommunications services. They were, and are, therefore, not subject to any of the content regulations that apply to commercial media. There is an additional background complexity to the situation in Australia: the Australia United States Free Trade Agreement (AUSFTA). While most expert opinion agrees that there is some scope within the AUSFTA for contemplating the application of Australian content requirements on what were called, when the agreement was settled back in 2004, ‘interactive audio and/or video services’, the political stakes of inviting a US trade backlash would be high.

But the issue of SVOD services sitting outside content regulation is changing. In 2018, the European Parliament updated regulation to require 30% of the catalogue of SVOD services on member countries to be European content. One of the most assertive regulatory actors in the EU, Germany, requires streaming services to contribute 2.5% of their revenue to the country’s subsidy system to support German production (Roxborough, 2018).

In Canada, a root and branch, holistic review of the broadcasting and telecommunications legislative frameworks has in January 2020 brought down its final report addressing reform principles (Broadcasting and Telecommunications Legislative Review Panel (BTLRP), 2020). This report has the virtue of having had to build from first principles the rationale for broadcasting and telecommunications reform. This is in contrast to EU actions which, having already developed extensive regulatory architecture applicable and in action in relation to the major US platforms, is extending and adapting rather than starting afresh, as *Canada’s Communications Future: Time to Act* is. It proposes to radically cut through decades of policy distinctions and exclusions in asserting that

This new model would bring all those providing media content services to Canadians – whether online or through conventional means, whether foreign or domestic, whether or not they have a place of business in Canada – within the scope of the Broadcasting Act and under the jurisdiction of the CRTC. (BTLRP, 2020: 11)

While maintaining flexibility as to the level of contribution, it includes every entity in an omnibus undertaking: ‘We recommend that all media content undertakings that benefit from the Canadian media communications sector contribute to it in an equitable manner: Undertakings that carry out like activities should have like obligations, regardless of where they are located’ (BTLRP, 2020: 143). This includes: curation (Prime Video, Netflix, Spotify and Canadian entities); aggregation (cable cos, MSN News, Google News and Apple News) and sharing (YouTube and Facebook).

In Australia, the situation is fluid and complex. Before COVID-19, there had been policy stasis for years. However, what had happened was the extension of the Location and Post, Digital and Visual Effects (PDV) Offsets to SVOD services (Fifield, 2019) to complement the already available Producer Offset and the 4-year AU$140 million Location Incentive available from July 2019. This was a potentially substantial ‘carrot’ that could precede at least a threatened regulatory ‘stick’. Both Netflix and Apple utilised the extension within months, bringing their respective *Shantaram* and *Clickbait* productions to film in Victoria (Film Victoria, 2019a, 2019b).

Some of the complexity of the regulatory challenge is seen when we consider that the ACCC Digital Platforms Inquiry is a 600+ page report setting an international gold standard of what assertive national jurisdictions could or should do when faced with the major social (including privacy), competition and public interest issues which particularly Google and Facebook pose for society and traditional media. There is nothing explicit in the report on SVODs, and only three mentions of Netflix – none of them in any way substantive. But it has been used not only to move to threaten mandatory ‘terms of trade’ between news media and Google and Facebook, but as a trigger for ramping up the significance of the cultural issue of sustainability of Australian and children’s content on screen media using the report’s recommendation for a ‘harmonised framework’ for media reform – even though this recommendation (Rec 6; ACCC, 2019: 15) contains nothing directly to do with SVOD services.

This would suggest that the government’s strategy is to transfer the high level of now-near global advocacy for reigning in the influence of the two major platforms in the social, competition and public interest fields and apply it to the cultural field to regulate SVOD services. But the opposite is just as likely.

‘Regulated free-to-air broadcasters are competing with unregulated digital platforms and video streaming services. It has been evident for some time – and the COVID-19 crisis has made it even more obvious – that this is not sustainable’, the communications minister recently noted (Fletcher as cited by Karp, 2020). ‘These arrangements threaten the sustainability of television broadcasters – and in turn the sustainability of the film and television content production sector’.

Government threatens ‘unregulated digital platforms and video streaming services’, but all the actions so far have been to deregulate and subsidise commercial broadcasters, now faced with a major acceleration of their loss of advertising base, and eyeballs to the streamers. The test will be whether anything on the side of regulating ‘unregulated digital platforms and video streaming services’ will arise from the fast-track options paper (ACMA and Screen Australia, 2020), apart from more help to commercial broadcasters. The Options Paper lays out four options: no change, minimal, significant and deregulation. It will be a test of local production industry advocacy to move the dial away from the government’s (hitherto incremental) deregulatory bent.

The situation going forward is complex because powerful local media with predominant interests in news and information want heavy, experimental, first-in-the-world regulation of Google and Facebook and the government has promised it: the development by the ACCC of a mandatory code requiring these two platforms to share advertising revenues with news media companies for content they created which the platforms are distributing. On the other hand, local media with predominant interests in entertainment (there are of course crossovers between the two) want radical deregulation of their Australian content obligations, and the government is ‘temporarily’ trialling it.

If there is to be a future for Australian content regulation on commercial television services, there has to be some parity established, with the imposition on international SVOD services of minimum Australian content requirements as well as facilitation of its discoverability. That is what is happening in Europe and Canada. That is what should happen here.

## Conclusion

We have offered a ‘screen-ecological’ approach that attempts to grasp the cultural and industrial dynamics on both the supply (production and business strategy) and demand (consumption) sides of the impact of multinational SVOD services in Australia. Such an approach emphasises the complex, conflictual and interdependent factors that must be taken into consideration in any holistic assessment of Netflix (and other multinational SVODs) in the Australian screen ecology. A stalled policy response to the challenge of unregulated SVOD services has been mightily shaken up when the government, under crisis conditions sparked by the global pandemic, ‘temporarily’ suspended Australian content regulations, versions of which have been in place since the 1960s.

Netflix’s model of internationalisation is very different from previous models media scholars and policy makers are used to. If we do not learn how to read and respond to Netflix’s model of internationalisation productively, it will have implications for both the maximisation of opportunities for the production industry out of the presence of international streamers in Australia, and for wider community support for incentives for, and potential future regulation of, the streamers here now and yet to arrive.

## References

[bibr1-1329878X20941173] ACCC (2019) Digital platforms inquiry – final report. June. Available at: https://www.accc.gov.au/system/files/Digital%20platforms%20inquiry%20-%20final%20report.pdf (accessed 26 July 2019).

[bibr2-1329878X20941173] ACMA (2020) Communications report 2018-19. Available at: https://www.acma.gov.au/sites/default/files/2020-02/Communications%20report%202018-19.pdf (accessed 27 February 2020).

[bibr3-1329878X20941173] ACMA and Screen Australia (2020) Supporting Australian stories on our screens. Options Paper, 15 April. Available at: https://www.communications.gov.au/have-your-say/supporting-australian-stories-our-screens-options-paper (accessed 15 April 2020).

[bibr4-1329878X20941173] AlphaBeta (2016) Bigger picture: the new age of screen content. Available at: https://www.alphabeta.com/wp-content/uploads/2016/12/Google_Bigger-Picture-Report_Dec2016.pdf (accessed 20 May 2020).

[bibr5-1329878X20941173] Ampere Analysis (2019) Ampere analysis: Amazon targets Australian market. Broadband TV News, 2 October. Available at: https://www.broadbandtvnews.com/2019/10/02/ampere-analysis-amazon-targets-australian-market/ (accessed 20 May 2020).

[bibr6-1329878X20941173] Ball M (2018) How the paradox of the term ‘original series’ explains the video industry (Netflix misunderstandings, Pt. 4). REDEF, 27 August. Available at: https://redef.com/original/how-the-paradox-of-the-phrase-original-series-explains-the-video-industry-netflix-misunderstandings-pt-4 (accessed 20 May 2020).

[bibr7-1329878X20941173] Bastow C (2016) No activity: Patrick Brammall and Darren Gilshenan deliver Australian farce at its finest. The Guardian, 26 October. Available at: https://www.theguardian.com/tv-and-radio/2016/oct/26/no-activity-patrick-brammall-and-darren-gilshenan-deliver-australian-farce-at-its-finest (accessed 8 April 2020).

[bibr8-1329878X20941173] Blackiston H (2019a) Amazon reboots Packed to the Rafters as its first original Australian drama. Mumbrella, 5 December. Available at: https://mumbrella.com.au/amazon-reboots-packed-to-the-rafters-as-its-first-original-australian-drama-609452 (accessed 20 May 2020).

[bibr9-1329878X20941173] Blackiston H (2019b) Stan CEO Mike Sneesby says Disney+ launch is ‘going to be terrific for everyone. Mumbrella, 18 November. Available at: https://mumbrella.com.au/stan-ceo-mike-sneesby-says-disney-launch-is-going-to-be-terrific-for-everyone-606887 (accessed 20 May 2020).

[bibr10-1329878X20941173] Boseley M (2020) Four Chris Lilley shows removed from Netflix Australia. The Guardian, 11 June. Available at: https://www.theguardian.com/tv-and-radio/2020/jun/11/four-chris-lilley-shows-removed-from-netflix-australia-library?utm_term=RWRpdG9yaWFsX0d1YXJkaWFuVG9kYXlBVVMtMjAwNjE0&utm_source=esp&utm_medium=Email&CMP=GTAU_email&utm_campaign=GuardianTodayAUS (accessed 11 June 2020).

[bibr11-1329878X20941173] Boyd C (2020) Film review: true history of the Kelly Gang. Screen Hub, 24 January. Available at: https://www.screenhub.com.au/news-article/reviews/digital/chris-boyd/film-review-true-history-of-the-kelly-gang-259491 (accessed 8 April 2020).

[bibr12-1329878X20941173] Bradshaw P (2019) True history of the Kelly Gang review – brutal portrait of the outback outlaw. The Guardian, 13 September. Available at: https://www.theguardian.com/film/2019/sep/13/true-history-of-the-kelly-gang-review-justin-kurzel (accessed 8 April 2020).

[bibr13-1329878X20941173] Broadcasting and Telecommunications Legislative Review Panel (BTLRP) (2020) Canada’s communications future: time to act. Final report, January. Available at: https://www.ic.gc.ca/eic/site/110.nsf/vwapj/BTLR_Eng-V3.pdf/$file/BTLR_Eng-V3.pdf (accessed 30 January 2020).

[bibr14-1329878X20941173] Buckmaster L (2016) Wolf Creek TV series review – Mick Taylor meets his match in outback slasher spin-off. The Guardian, 13 May. Available at: https://www.theguardian.com/tv-and-radio/2016/may/13/wolf-creek-tv-series-review-mick-taylor-outback-slasher (accessed 8 April 2020).

[bibr15-1329878X20941173] Buckmaster L (2017) In the wake of Milo Yiannopoulos’ tour, do we really need another Romper Stomper? The Guardian, 6 December. Available at: https://www.theguardian.com/tv-and-radio/2017/dec/06/in-the-wake-of-milo-yiannopoulos-tour-do-we-really-need-another-romper-stomper (accessed 8 April 2020).

[bibr16-1329878X20941173] Buckmaster L (2018a) Pine Gap review – lots of Yakkety Yak and occasional scenes of bonking. The Guardian, 12 October. Available at: https://www.theguardian.com/tv-and-radio/2018/oct/12/pine-gap-review-lots-of-yakkety-yak-and-occasional-scenes-of-bonking (accessed 8 April 2020).

[bibr17-1329878X20941173] Buckmaster L (2018b) The second review – Rachael Blake ventures to dark places in steamy thriller. The Guardian, 4 July. Available at: https://www.theguardian.com/film/2018/jul/04/the-second-review-rachael-blake-ventures-to-dark-places-in-steamy-thriller (accessed 8 April 2020).

[bibr18-1329878X20941173] Buckmaster L (2019a) Chris Lilley’s Lunatics review: unfunny, boring and utterly unrelenting. The Guardian, 20 April. Available at: https://www.theguardian.com/tv-and-radio/2019/apr/20/chris-lilleys-lunatics-review-unfunny-boring-and-utterly-unrelenting (accessed 8 April 2020).

[bibr19-1329878X20941173] Buckmaster L (2019b) The Commons review – eerily plausible and uncomfortably timely climate crisis drama. The Guardian, 26 December. Available at: https://www.theguardian.com/tv-and-radio/2019/dec/26/the-commons-review-eerily-plausible-and-uncomfortably-timely-climate-crisis-drama (accessed 8 April 2020).

[bibr20-1329878X20941173] Buckmaster L (2019c) The Gloaming review – shades of Twin Peaks in fog-swamped crime drama. The Guardian, 30 December. Available at: https://www.theguardian.com/tv-and-radio/2019/dec/30/the-gloaming-review-shades-of-twin-peaks-in-fog-swamped-drama (accessed 8 April 2020).

[bibr21-1329878X20941173] Burke J (2015) Quick bites – pay-TV. The Australian, 17 October, p. 25.

[bibr22-1329878X20941173] Burke J (2017) All aboard a bus bound for mayhem and Mick. The Australian, 18 December, p. 18.

[bibr23-1329878X20941173] Clarke S (2019a) A look at Netflix’s ever-increasing physical footprint in international territories. Variety, 7 November. Available at: https://variety.com/2019/tv/news/netflix-international-territories-1203395845/ (accessed 7 November 2019).

[bibr24-1329878X20941173] Clarke S (2019b) Berlin: Netflix talks European content, finding new voices. Variety, 13 February. Available at: https://variety.com/2019/tv/news/netflix-more-international-series-berlin-drama-series-days-1203138365/ (accessed 15 February 2019).

[bibr25-1329878X20941173] Cunningham S (2018/19) Follow this. InterMEDIA 46(4): 12–15.

[bibr26-1329878X20941173] Dermody S Jacka E (1987) The Screening of Australia. Sydney, NSW, Australia: Currency Press.

[bibr27-1329878X20941173] Duke J (2018) Stan boss backs Aussie content but says quotas ‘not required’. The Sydney Morning Herald, 6 June. Available at: https://www.smh.com.au/business/companies/stan-boss-backs-aussie-content-but-says-quotas-not-required-20180606-p4zjsd.html (accessed 20 May 2020).

[bibr28-1329878X20941173] Enker D (2018) Netflix wades into Australian waters with epic supernatural mystery Tidelands. The Age, 29 November. Available at: https://www.theage.com.au/entertainment/tv-and-radio/netflix-wades-into-australian-waters-with-epic-supernatural-mystery-tidelands-20181129-h18iy1.html?btis (accessed 8 April 2020).

[bibr29-1329878X20941173] European Parliament (2018) New rules for audiovisual media services approved by parliament. Press Release, 2 October. Available at: https://www.europarl.europa.eu/news/en/press-room/20180925IPR14307/new-rules-for-audiovisual-media-services-approved-by-parliament (accessed 15 May 2020).

[bibr30-1329878X20941173] Fernandes T (2019) ACCC digital platforms inquiry – submission in response to the final report. Motion Picture Association – International, 12 September. 1–7.

[bibr31-1329878X20941173] Fifield M (2019) More opportunities for Australia’s world-class screen industry. Press Release, 11 April. Available at: https://www.mitchfifield.com/2019/04/more-opportunities-for-australias-world-class-screen-industry/ (accessed 11 April 2019).

[bibr32-1329878X20941173] Film Victoria (2019a) Epic bestseller Shantaram to film in Victoria. Press Release, 15 August. Available at: https://www.film.vic.gov.au/news/epic-bestseller-shantaram-to-film-in-victoria (accessed 20 May 2020).

[bibr33-1329878X20941173] Film Victoria (2019b) Netflix partners with Victorian creators to bring Clickbait to Melbourne. Press Release, 26 August. Available at: https://www.film.vic.gov.au/news/netflix-partners-with-victorian-creators-to-bring-clickbait-to-melbourne (accessed 20 May 2020).

[bibr34-1329878X20941173] Fletcher P (2020) Speech to the International Institute of Communications: the harmonised framework – digital platforms & the media. 21 February. Available at: https://www.paulfletcher.com.au/portfolio-speeches/speech-to-the-international-institute-of-communications-the-harmonised-framework (accessed 21 February 2020).

[bibr35-1329878X20941173] Galea M (2019) Why ‘The Other Guy’ is the most legit TV show about Aussie millennials. Pedestrian, 13 December. Available at: https://www.pedestrian.tv/entertainment/the-other-guy-season-2-2/ (accessed 8 April 2020).

[bibr36-1329878X20941173] Galea M (2020) The critics are raving about ‘True History of the Kelly Gang’, here’s why you’ll love it too. Pedestrian, 27 January. Available at: https://www.pedestrian.tv/entertainment/true-history-of-the-kelly-gang-review/ (accessed 8 April 2020).

[bibr37-1329878X20941173] Gaunson S (2020) True history of the Kelly Gang review: an unheroic portrait of a violent, unhinged, colonial punk. The Conversation, 9 January. Available at: https://theconversation.com/true-history-of-the-kelly-gang-review-an-unheroic-portrait-of-a-violent-unhinged-colonial-punk-128463 (accessed 8 April 2020).

[bibr38-1329878X20941173] Grece C (2018) Films in VOD catalogues – origin, circulation and age – edition 2018. Strasbourg: European Audiovisual Observatory. Available at: https://rm.coe.int/films-in-vod-catalogues-origin-circulation-and-age-edition-2018/168094627a (accessed 15 May 2020).

[bibr39-1329878X20941173] Groves D (2019) Stan wants more of the other guy. C21 Media, 23 January. Available at: https://www.c21media.net/stan-finds-the-other-guy/ (accessed 8 April 2020).

[bibr40-1329878X20941173] Groves D (2020) Stan primes ‘True History of the Kelly Gang’ with limited cinema release. Inside Film, 13 January. Available at: https://www.if.com.au/stan-primes-true-history-of-the-kelly-gang-with-limited-cinema-release/ (accessed 8 April 2020).

[bibr41-1329878X20941173] Hastings R (2019) New York Times dealbook conference. Interview, 6 November. Available at: https://youtu.be/7V6FFeZdFz4 (accessed 8 November 2019).

[bibr42-1329878X20941173] Heritage S (2019) Lunatics is nasty and grotesque. Chris Lilley’s career is shot. The Guardian, 23 April. Available at: https://www.theguardian.com/tv-and-radio/2019/apr/23/lunatics-is-nasty-and-grotesque-chris-lilleys-career-is-shot-surely (accessed 8 April 2020).

[bibr43-1329878X20941173] Houston M (2018) Tidelands. The Age, 16 December, p. 16.

[bibr44-1329878X20941173] Idato M (2017) Networking. The Age, 21 September, p. 2.

[bibr45-1329878X20941173] Karp P (2020) Local content quotas suspended in $54m package for Australia’s coronavirus-hit media. The Guardian, 15 April. Available at: https://www.theguardian.com/media/2020/apr/15/local-content-quotas-suspended-in-54m-package-for-australias-coronavirus-hit-media (accessed 15 April 2020).

[bibr46-1329878X20941173] Kidman A (2020) Apple’s iPhone Australian market share grows as Huawei crashes. Gizmodo, 29 January. Available at: https://www.gizmodo.com.au/2020/01/apples-iphone-australian-market-share-grows-as-huawei-crashes/ (accessed 8 April 2020).

[bibr47-1329878X20941173] LaMarco P (2018) REVIEW: Netflix’s ‘Pine Gap’ is a dull and sluggish attempt at a thriller. U-Wire, 9 December. Available at: https://dailyfreepress.com/2018/12/09/review-netflixs-pine-gap-is-a-dull-and-sluggish-attempt-at-a-thriller/ (accesed 8 April 2020).

[bibr48-1329878X20941173] Lobato R Ewing S (2014) Unlocking the geoblocks: Australians embrace VPNs. The Conversation, 2 October. Available at: https://theconversation.com/unlocking-the-geoblock-australians-embrace-vpns-32373 (accessed 15 May 2020).

[bibr49-1329878X20941173] Lobato R Scarlata A (2019) Australian Content in SVOD Catalogs: Availability and Discoverability –2019 Edition. Report. Melbourne: RMIT University. Available: https://apo.org.au/node/264821 (accessed 29 October 2019).

[bibr50-1329878X20941173] Lodge G (2019) Toronto film review: ‘True History of the Kelly Gang’. Variety, 6 September. Available at: https://variety.com/2019/film/reviews/true-history-of-the-kelly-gang-review-1203324807/ (accessed 8 April 2020).

[bibr51-1329878X20941173] Lotz A (2020) Netflix 30 Q&A. 29 June. Available at: http://www.amandalotz.com/netflix-30-qa/2020/6/29/q8-why-is-competition-the-wrong-frame-for-understanding-the-relationship-between-svods-and-ad-supported-linear-television (accessed 29 June 2020).

[bibr52-1329878X20941173] Ma W (2018) Netflix’s Tidelands is an embarrassing mess’. news.com.au, 12 December. Available at: https://www.news.com.au/entertainment/tv/tv-shows/netflixs-first-australian-original-series-tidelands-is-lightweight-and-underwhelming/news-story/5c75ae5aa3ecc3e9c55d5ac53be6aeb7 (accessed 8 April 2020).

[bibr53-1329878X20941173] Ma W (2019a) The Commons feels less like drama and more like prophecy. news.com.au, 26 December. Available at: https://www.news.com.au/entertainment/tv/tv-shows/the-commons-feels-less-like-drama-and-more-like-prophecy/news-story/170740414347d7247405a60f5c655b69 (accessed 8 April 2020).

[bibr54-1329878X20941173] Ma W (2019b) Worst part about Lilley’s Netflix series. news.com.au, 19 April. Available at: https://www.news.com.au/entertainment/tv/tv-shows/chris-lilleys-lunatics-not-that-outrageous-not-that-funny/news-story/58bd6284c22f234c05e481acd9cf1a8e (accessed 8 April 2020).

[bibr55-1329878X20941173] Ma W (2020) The Gloaming wants us to confront our past. news.com.au, 2 January. Available at: https://www.news.com.au/entertainment/tv/tv-shows/the-gloaming-wants-us-to-confront-our-past/news-story/c046f12cf1724f85060b88efcb18aa7e (accessed 8 April 2020).

[bibr56-1329878X20941173] McDonnell J (2019) Foxtel now app scrapped in favour of GO platform. AdNews, 16 September. Available at: https://www.adnews.com.au/news/foxtel-now-app-scrapped-in-favour-of-go-platform (accessed 15 May 2020).

[bibr57-1329878X20941173] Masige S (2019) Netflix is raising the price of its premium plan … . Business Insider Australia, 3 October. Available at: https://www.businessinsider.com.au/netflix-premium-plan-increase-australia-2019-10 (accessed 3 November 2019).

[bibr58-1329878X20941173] Meade A (2020) Nine could cut Ashes and T20 World Cups from free-to-air TV as profits fall. The Guardian, 26 February. Available at: https://www.theguardian.com/media/2020/feb/26/nine-could-cut-ashes-and-t20-world-cups-from-free-to-air-tv-as-profits-fall (accessed 26 February 2020).

[bibr59-1329878X20941173] Mediaweek (2020) Nine’s Hugh Marks reveals initiatives to ride out market uncertainty. 31 March. Available at: https://www.mediaweek.com.au/nines-hugh-marks-reveals-initiatives-to-ride-out-market-uncertainty/ (accessed 31 March 2020).

[bibr60-1329878X20941173] Moran R (2017) The naked truth. The Age, 10 August, p. 3.

[bibr61-1329878X20941173] Morris A (2019) TV: the terrifying near-future of ‘The Commons’ feels all too real. Screen Hub, 19 December. Available at: https://www.screenhub.com.au/news-article/reviews/television/anthony-morris/tv-the-terrifying-near-future-of-the-commons-feels-all-too-real-259477 (accessed 8 April 2020).

[bibr62-1329878X20941173] Netflix Investors (2019) 2019 quarterly earnings, Q3. Letter to Shareholders, 16 October. Available at: https://s22.q4cdn.com/959853165/files/doc_financials/quarterly_reports/2019/q3/FINAL-Q3-19-Shareholder-Letter.pdf (accessed 16 October 2019).

[bibr63-1329878X20941173] Nsenduluka B (2019) ‘Just cancelled my subscription’: outraged fans slam Chris Lilley for wearing ‘blackface’ in trailer for new Netflix show Lunatics. Daily Mail Australia, 11 April. Available at: https://www.dailymail.co.uk/tvshowbiz/article-6910285/Outraged-fans-slam-Chris-Lilley-wearing-blackface-trailer-new-Netflix-Lunatics.html (accessed 8 April 2020).

[bibr64-1329878X20941173] Potter A (2018) Creating children’s television for SVODs: the alignment of global production practices with national screen policies in the Netflix original Bottersnikes and Gumbles. Media Industries 5(2): 111–127.

[bibr65-1329878X20941173] Powell E (2019) Chris Lilley Lunatics roles sparks ‘blackface’ row as Netflix comedy faces Twitter backlash. Evening Standard, 11 April. Available at: https://www.msn.com/en-gb/entertainment/tv/chris-lilley-lunatics-role-sparks-blackface-row-as-netflix-comedy-faces-twitter-backlash/ar-BBVPM0G (accessed 8 April 2020).

[bibr66-1329878X20941173] Quinn K (2017) Politics with real punch. Canberra Times, 30 December, p. 16.

[bibr67-1329878X20941173] Quinn K (2018) ‘Bloom covers the waterfront’: Jacki Weaver on Stan’s ‘gothic fairytale’. The Sydney Morning Herald, 27 December. Available at: https://www.smh.com.au/entertainment/tv-and-radio/bloom-covers-the-waterfront-jacki-weaver-on-stan-s-gothic-fairytale-20181220-p50nep.html (accessed 8 April 2020).

[bibr68-1329878X20941173] Razer H (2018) The ABC’s Pine Gap is a stinker. Daily Review, 11 October. Available at: https://dailyreview.com.au/abcs-pine-gap-stinker/ (accessed 8 April 2020).

[bibr69-1329878X20941173] Rickett O (2015) How ‘Netflix and chill’ became code for casual sex. The Guardian, 30 September. Available at: https://www.theguardian.com/media/shortcuts/2015/sep/29/how-netflix-and-chill-became-code-for-casual-sex (accessed 15 May 2020).

[bibr70-1329878X20941173] Rooney D (2019) ‘True history of the Kelly Gang’: film review| TIFF 2019. The Hollywood Reporter, 6 September. Available at: https://www.hollywoodreporter.com/review/true-history-kelly-gang-review-tiff-2019-1235961 (accessed 8 April 2020).

[bibr71-1329878X20941173] Roxborough S (2018) Netflix criticizes European content quota. The Hollywood Reporter, 17 October. Available at: https://www.hollywoodreporter.com/news/netflix-criticizes-european-content-quota-1152970 (accessed 15 May 2020).

[bibr72-1329878X20941173] Roy Morgan (2016) More Australians now have SVOD than Foxtel. Press Release, 8 September. Available at: http://www.roymorgan.com/findings/6957-svod-overtakes-foxtel-pay-tv-in-australia-august-2016-201609081005 (accessed 10 June 2020).

[bibr73-1329878X20941173] Roy Morgan (2019) Almost 14 million Australians have subscription or pay TV. Press Release, 1 July. Available at: http://www.roymorgan.com/findings/8036-svod-netflix-foxtel-stan-fetch-youtube-amazon-pay-tv-may-2019-201907010501 (accessed 10 June 2020).

[bibr74-1329878X20941173] Roy Morgan (2020) Disney Plus attracts over 1.8 million in first 3 months. Press Release, 31 March. Available at: http://www.roymorgan.com/findings/8348-subscription-pay-tv-services-february-2020-202003300559 (accessed 10 June 2020).

[bibr75-1329878X20941173] Samios Z Hunter F (2020) Seven halts children’s production in Australian content quota protest. The Sydney Morning Herald, 26 February. Available at: https://www.smh.com.au/business/companies/seven-halts-children-s-production-in-australian-content-quota-protest-20200225-p5445r.html (accessed 26 February 2020).

[bibr76-1329878X20941173] Screen Australia (2019) Screen Australia drama report 2018/19. 31 October. Available at: https://www.screenaustralia.gov.au/getmedia/08d8518b-867b-4f61-8c2e-ebd10f0dc3a4/Drama-Report-2018-2019.pdf (accessed 15 May 2020).

[bibr77-1329878X20941173] Screen Queensland (2017) Queensland snares the first Netflix series to shoot in Australia. Press Release, 16 May. Available at: https://screenqueensland.com.au/sq-news/queensland-snares-the-first-netflix-series-to-shoot-in-australia/ (accessed 17 May 2017).

[bibr78-1329878X20941173] Seven West Media (2018) ACCC digital platforms inquiry. Submission, 23 April. Available at: https://www.accc.gov.au/system/files/Seven%20West%20Media%20%28April%202018%29.pdf (accessed 15 May 2020).

[bibr79-1329878X20941173] Slessor C Bogle I (2018) Netflix series filmed in Adelaide ‘the beginning’ of content creation for streaming services. ABC News, 5 April. Available at: https://www.abc.net.au/news/2018-04-05/pine-gap-netflix-drama-series-filmed-in-sa/9621320#:~:targetText=A%20new%20Netflix%20series%20being,the%20South%20Australian%20Film%20Corporation (accessed 8 April 2020).

[bibr80-1329878X20941173] Sneesby M (2019) In conversation with Stan: Mike Sneesby. In: Panel, screen forever conference, Melbourne, VIC, Australia, 13 November.

[bibr81-1329878X20941173] Spangler T (2018) Netflix content boss Ted Sarandos downplays looming threat from Disney, WarnerMedia. Variety, 3 December. Available at: https://variety.com/2018/digital/news/netflix-ted-sarandos-threat-disney-warnermedia-streaming-1203078374/ (accessed 15 May 2020).

[bibr82-1329878X20941173] Spangler T (2019a) Netflix ranked as No. 1 fastest-growing U.S. brand in 2019. Variety, 28 March. Available at: https://variety.com/2019/digital/news/netflix-brand-value-ranking-2019-1203175244/ (accessed 24 April 2019).

[bibr83-1329878X20941173] Spangler T (2019b) The streaming wars’ other battlefront: user experience as important as content, survey finds. Variety, 23 April. Available at: https://variety.com/2019/digital/news/streaming-video-usability-research-consumer-perceived-value-pwc-1203194587/ (accessed 24 April 2019).

[bibr84-1329878X20941173] Stan Australia (2019) Australia’s biggest summer line-up of original productions| ANNOUNCEMENT| This summer on Stan. YouTube, 16 November. Available at: https://www.youtube.com/watch?v=qN4v2AyOQ8s&feature=youtu.be (accessed 8 April 2020).

[bibr85-1329878X20941173] Turner A (2018) Tidelands struggles to stay afloat in its first series. The Conversation, 18 December. Available at: https://theconversation.com/tidelands-struggles-to-stay-afloat-in-its-first-series-108751 (accessed 8 April 2020).

[bibr86-1329878X20941173] Turner G (2018) Netflix and the reconfiguration of the Australian television market. Media Industries 5(2): 129–142.

[bibr87-1329878X20941173] Turner G (2019a) Approaching the cultures of use: Netflix, disruption and the audience. Critical Studies in Television 14(2): 222–232.

[bibr88-1329878X20941173] Turner G (2019b) Television studies, we need to talk about ‘Binge-Viewing’. Television & New Media, Online, 26 September, p. 1–13.

[bibr89-1329878X20941173] Valentish J (2020) Matt Okine’s The Other Guy lays out the ethical gymnastics of art imitating life … imitating art. The Guardian, 5 January. Available at: https://www.theguardian.com/tv-and-radio/2020/jan/05/matt-okines-the-other-guy-lays-out-the-ethical-gymnastics-of-art-imitating-life-imitating-art (accessed 8 April 2020).

[bibr90-1329878X20941173] Verhoeven D (2014) Film, video, DVD and online delivery. In: Cunningham S Turnbull S (eds) The Media and Communications in Australia. Crows Nest, NSW, Australia: Allen & Unwin, pp. 151–171.

[bibr91-1329878X20941173] Vourlias C (2020) Netflix looks to dispel myths in Berlin as it ramps up European production. Variety, 26 February. Available at: https://variety.com/2020/digital/global/netflix-european-production-1203516140/ (accessed 26 February 2020).

[bibr92-1329878X20941173] White P Kanter J (2019) Netflix reveals most popular international titles of 2019: Madeleine McCann True-Crime Doc Tops UK, ‘Murder Mystery’ leads in Australia. Deadline, 30 December. Available at: https://deadline.com/2019/12/netflix-reveals-most-popular-international-titles-of-2019-madeleine-mccann-true-crime-doc-tops-uk-murder-mystery-leads-in-australia-1202818375/ (accessed 15 May 2020).

